# ANXIETY, COGNITIVE FUNCTION, CHANGE, AND IMPAIRMENT AMONG DIVERSE OLDER HISPANICS/LATINOS

**DOI:** 10.1093/geroni/igac059.2823

**Published:** 2022-12-20

**Authors:** Roberto Gonzalez-Prado, Alejandra Morlett Paredes, Wassim Tarraf, Ariana Stickel, Fernando Testai, Linda Gallo, Frank Pendedo, Hector González

**Affiliations:** San Diego State University, San Diego, California, United States; University of California, San Diego, San Diego, California, United States; Wayne State University, Detroit, Michigan, United States; San Diego State University, San Diego, California, United States; University of Illinois, Chicago, Chicago, Illinois, United States; San Diego State University, San Diego, California, United States; University of Miami, Coral Gables, Florida, United States; University of California, San Diego, San Diego, California, United States

## Abstract

**Introduction:**

Anxiety and depression often co-occur and may be risk factors for neurodegenerative diseases such as Alzheimer’s disease and related dementias. We examined associations between symptoms of anxiety and cognitive change and impairment in middle-aged and older Hispanics/Latinos. Method: 6,162 participants aged ≥50 (mean age=63.4, SD=±8.2 years) were enrolled in the Study of Latinos-Investigations of Neurocognitive Aging study (SOL-INCA), an ancillary to the Hispanic Community Health Study/Study of Latinos (HCHS/SOL). Exposures included the Spielberger State-Trait Anxiety Inventory 10-item (STAI-10) and the Center for Epidemiology Scale for Depression 10-item (CESD-10) at HCHS/SOL Visit 1 (2008–2011). Outcomes include cognitive tests at Visit 1 and 2 about 7 years later in SOL-INCA and mild cognitive impairment diagnosis (MCI; NIA-AA diagnostic criteria). Separate models were created to examine associations between cognition and anxiety adjusting for demographic variables, antianxiety medication, and depressive symptoms.

**Results:**

After adjusting for demographic variables and medication use, STAI-10 scores were associated with Visit 2 cognitive function (p < 0.05) and MCI (p < 0.01), but not with changes in cognitive function over time. After adjusting for depressive symptoms, most effects of anxiety were attenuated, with exception of B-SEVLT-Recall (p < 0.05). Discussion: Anxiety symptoms were associated with lower memory function 7 years later among diverse older Hispanics/Latinos. Anxiety was associated with 7-year cognitive change and MCI, but those associations were explained by depressive symptoms and other covariables. Future research should examine cognition in relation to anxiety and depression since the two commonly co-occur.

